# Functional Vascular Tissue Engineering Inspired by Matricellular Proteins

**DOI:** 10.3389/fcvm.2019.00074

**Published:** 2019-05-31

**Authors:** Aneesh K. Ramaswamy, David A. Vorp, Justin S. Weinbaum

**Affiliations:** ^1^Department of Bioengineering, University of Pittsburgh, Pittsburgh, PA, United States; ^2^McGowan Institute for Regenerative Medicine, University of Pittsburgh, Pittsburgh, PA, United States; ^3^Department of Surgery, University of Pittsburgh, Pittsburgh, PA, United States; ^4^Department of Cardiothoracic Surgery, University of Pittsburgh, Pittsburgh, PA, United States; ^5^Department of Chemical and Petroleum Engineering, University of Pittsburgh, Pittsburgh, PA, United States; ^6^Department of Pathology, University of Pittsburgh, Pittsburgh, PA, United States

**Keywords:** collagen, elastin, extracellular matrix, endothelial cells, smooth muscle cells, thrombosis

## Abstract

Modern regenerative medicine, and tissue engineering specifically, has benefited from a greater appreciation of the native extracellular matrix (ECM). Fibronectin, collagen, and elastin have entered the tissue engineer's toolkit; however, as fully decellularized biomaterials have come to the forefront in vascular engineering it has become apparent that the ECM is comprised of more than just fibronectin, collagen, and elastin, and that cell-instructive molecules known as matricellular proteins are critical for desired outcomes. In brief, matricellular proteins are ECM constituents that contrast with the canonical structural proteins of the ECM in that their primary role is to interact with the cell. Of late, matricellular genes have been linked to diseases including connective tissue disorders, cardiovascular disease, and cancer. Despite the range of biological activities, this class of biomolecules has not been actively used in the field of regenerative medicine. The intent of this review is to bring matricellular proteins into wider use in the context of vascular tissue engineering. Matricellular proteins orchestrate the formation of new collagen and elastin fibers that have proper mechanical properties—these will be essential components for a fully biological small diameter tissue engineered vascular graft (TEVG). Matricellular proteins also regulate the initiation of thrombosis via fibrin deposition and platelet activation, and the clearance of thrombus when it is no longer needed—proper regulation of thrombosis will be critical for maintaining patency of a TEVG after implantation. Matricellular proteins regulate the adhesion, migration, and proliferation of endothelial cells—all are biological functions that will be critical for formation of a thrombus-resistant endothelium within a TEVG. Lastly, matricellular proteins regulate the adhesion, migration, proliferation, and activation of smooth muscle cells—proper control of these biological activities will be critical for a TEVG that recellularizes and resists neointimal formation/stenosis. We review all of these functions for matricellular proteins here, in addition to reviewing the few studies that have been performed at the intersection of matricellular protein biology and vascular tissue engineering.

## Introduction

Modern regenerative medicine, and tissue engineering specifically, has benefited from a greater appreciation of the native extracellular matrix (ECM). Fibronectin, collagen, and elastin have entered the tissue engineer's toolkit: peptides derived from fibronectin are routinely added to synthetic scaffolds to promote adhesion of seeded cells, high collagen content has been desirable for mechanically strong engineered tissues, and elastin has been an agreed-upon target for tissues that rely on recoil such as engineered blood vessels, skin, and lungs. It has become apparent that ECM-based biomaterials obtained by decellularization of native intestine, bladder, and heart tissues have a strong positive influence on *in vivo* regeneration. This cell-instructive potential includes the recruitment of host stem cells, modulation of the immune system, and promotion of cell differentiation to a beneficial phenotype for repair and later homeostasis. Notably, the ECM molecules present in these decellularized biomaterials go beyond just fibronectin, collagen, and elastin and include cell-instructive molecules known as matricellular proteins.

The term “matricellular” was coined by Paul Bornstein and members of his group in 1995. Of note, a special issue of Matrix Biology was published in the summer of 2014 to celebrate Paul Bornstein's legacy, and is recommended reading for more details on the evolution of this field. In brief, matricellular proteins are ECM constituents that contrast with the canonical structural proteins of the ECM in that their primary role is to interact with the cell. General observations of matricellular activity have included a modulatory effect on cell adhesion (hence their alternate name, “modulatory adhesion proteins”) and mouse phenotypes characterized by an altered response to injury. Of late, matricellular genes have been linked to diseases including connective tissue disorders, cardiovascular disease, and cancer. Despite the range of biological activities, this class of biomolecules has not been actively used in the field of regenerative medicine. The intent of this review is to bring these proteins (and their constitutive active peptides) into wider use in the context of vascular tissue engineering. A list of the proteins reviewed here can be found in Table 1.

**Table 1:** Matricellular proteins discussed in this review.

**Protein name**	**Col/Eln**	**Thr**.	**EC**	**SMC**
Aggrecan	X			
CCN1 (also Cyr61)	X		X	
CCN2 (also CTGF)	X		X	X
CCN3 (also Nov)			X	X
CCN4 (also Wisp-1)				X
CCN5 (also COMP)				X
Decorin	X		X	
Emilin-1	X	X		
Emilin-2		X		
Fibrillin-1	X		X	
Fibrillin-2	X			
Fibromodulin				X
Fibulin-1	X	X		X
Fibulin-2	X			
Fibulin-5	X			X
Galectin-1				X
Latent TGF-binding protein-1 (LTBP-1)	X			
Lumican	X		X	
Microfibril-associated glycoprotein-1 (MAGP-1)	X	X		X
Microfibril-associated glycoprotein-2 (MAGP-2)			X	X
Osteopontin (OPN, also bone sialoprotein I, BSP-1)				X
SPARC (also osteonectin)	X		X	
Syndecan-4	X			
Tenascin-C (TNC)				X
Tenascin-X (TNX)	X		X	
Thrombospondin-1 (TSP-1)		X	X	X
Thrombospondin-2 (TSP-2)		X	X	
Versican	X			
Vitronectin		X	X	

In the realm of vascular engineering, there are four key design parameters that must be met for a successful tissue engineered vascular graft (TEVG). First, the graft must have mechanical strength compatible with hemodynamics and local tissue deformation. Second, the implanted graft must present a non-thrombogenic luminal surface; that is, it will not induce activation of the host extrinsic or intrinsic clotting pathways or promote platelet adhesion and activation. Third, the luminal surface of the graft must support the formation and maintenance of an endothelial lining, with the endothelial cell source either an implanted graft or the host. Finally, the interior of the graft wall should be, or become, populated by cells; the most important cell type to populate the new media will be vascular smooth muscle cells. Of note, the smooth muscle cells that repopulate the graft must be restricted from over-proliferation, which could lead to graft blood flow restrictions by neointimal formation and eventual stenosis. In this review, we will examine how the biological activity of matricellular proteins could help vascular engineers achieve each of these four parameters for graft design.

## Matricellular Proteins Regulate the Production of Collagen and Elastin, the Key Molecular Determinants of Vascular Biomechanics

The compliance, elastic modulus, and burst strength of grafts have been primary criteria for success. In native vessels, the primary load-bearing proteins in the ECM are collagen and elastin, which combine to give the vessel a biphasic passive response to mechanical stretch (1). To mimic the native vessel, the presence of collagen, elastin, or synthetic substitutes for these molecules has been desirable in engineered grafts. In this section, we will focus on how matricellular proteins regulate the production of collagen and elastin in the context of biological grafts.

### Collagen Overview

In the vasculature, the primary collagens which contribute to mechanical strength are the fibrillar collagens, Type I and Type III. Two human genes, COL1A1 and COL1A2, encode the protein components of Type I collagen. In contrast, Type III collagen is produced from a single gene, COL3A1. After transcription and translation, the collagen chains are then post-translationally modified (by proteins such as members of the prolyl hydroxylase and lysyl oxidase (LOX) families) and wound into a collagen triple-helix, comprised of three protein chains per collagen fiber. Collagen fibers are then combined with a staggered overlap into a larger bundle, resulting in fiber cooperativity and a high overall tensile strength of the higher order structure.

### Collagen Transcription

Research into the transcriptional control of fibrillar collagens has benefited from the study of scarring in skin. Of note, one hallmark of scarring is the secretion of pro-inflammatory cytokines by immune cells during the wound healing response. The canonical signaling pathway for fibrosis involves transforming growth factor (TGF)-β1. After binding of TGF-β1 to its cellular receptor, the SMAD 2/3 signaling pathway is activated, leading to translocation of SMAD3/4 to the cell's nucleus. SMAD-binding enhancer sequences are found in the promoters for COL1A1 (2), COL1A2 (3), and COL3A1 (4). TGF-β1 functionality is primarily regulated in the vasculature by its binding to the matricellular proteins latent TGF-β binding protein (LTBP)-1 and−4 (5). A molecule with many overlapping functions to TGF-β1 in fibrosis, connective tissue growth factor (CTGF or CCN2) directly promotes collagen synthesis to support either new or healing vascular beds (6), and parallels collagen I expression in pericytes (7).

### Collagen Post-translational Modification

In Ehlers-Danlos Syndrome, defects in the vascular wall, particularly in collagen fibers, lead to vascular fragility. One key player in this process is the matricellular protein tenascin-X (TNX). TNX absence results in the loss of structural integrity of the vascular collagenous matrix and a reduction of collagen-processing enzyme efficacy (8). Proteoglycan syndecan-4 promotes post-translational collagen LOX modification directly via its extracellular domain ECsyn4, and indirectly via osteopontin (9). Additionally, stimulation of cardiac fibroblasts with recombinant lumican, a collagen-binding proteoglycan, increased LOX and matrix metalloproteinase-9 (10), leading to hypotheses about the role of lumican in myocardial fibrosis (11) and as a biomarker for acute aortic dissection (12).

### Collagen Fiber Assembly

Perhaps the greatest effect of matricellular proteins is on the assembly of collagen fibers, where they act as quality control regulators of collagen fiber size and stability. CCN family member 1 (CCN1), otherwise known as cysteine-rich angiogenic inducer 61 (CYR61), modulates collagen I stability through surface integrins in response to serum concentration levels of basic fibroblast growth factor (bFGF) and vascular endothelial growth factor (VEGF) (6). Secreted protein acidic and rich in cysteine (SPARC), or osteonectin, plays a vital role in binding and organizing collagen (13). SPARC primarily aggregates in vascular basement membranes, modulating procollagen processing and collagen interactions with cardiac fibroblasts (14) producing a series of smaller and more tightly organized collagen I fibrils (15).

Decorin is a member of the small leucine-rich proteoglycan family of matricellular proteins that sequesters TGF-(beta) activity, with glycoproteomic analysis showing decorin-derived peptide fragments present throughout the cardiac ECM (16). Decorin has been shown to influence collagen matrix organization and gel contraction, binding to collagens I-III, VI, and XIV (17) resulting in increased collagen fibril density and decreased diameter (18). The effects of decorin-derived peptides have been studied as a potential way of modulating collagen fibrillogenesis (19) and neointimal hyperplasia following balloon angioplasty (20, 21). Decorin in the context of aortic aneurysm has a complex role: decorin associates with a lowered risk of rupture in murine abdominal aortic aneurysm (22), but while decorin serves a protective role in early in murine aneurysm formation it also correlates strongly with macrophage matrix metalloproteinase (MMP)-9 expression and lesion location (23).

### Elastin Overview

In the vasculature, the property of passive recoil in response to pulsatile blood flow is provided by the elastic fiber. While other proteins are responsible for the assembly and biological activity of the elastic fiber, the primary protein component is elastin. A single elastin gene is present in the mammalian genome, and mutation or heterozygosity of the gene is linked to several human diseases including Williams Syndrome (24), cutis laxa (25), and supravalvular aortic stenosis (26). After transcription and translation, elastin is trafficked to the surface of the cell, where it is chaperoned to fibrillin microfibrils and assembled. Intra and inter-molecular crosslinking of elastin is then performed by members of the lysyl oxidase family, producing a resilient, and elastic macrostructure.

### Elastin Transcription

Like collagen, elastin can be upregulated by TGF-β signaling. Uniquely, TGF-β regulation of elastin message level occurs post-transcriptionally through stabilization of the messenger RNA (27).

One component of the elastic fiber, the matricellular protein microfibril-associated glycoprotein-1 (MAGP-1), activates latent TGF-β signaling directly (28), potentially presenting an opportunity for molecular engineers looking to encourage elastogenesis. MAGP-1 inhibits binding of LTBP-1 to fibrillin-1 and could fine tune concentration and deposition of the large latent TGF-β (LLC) complex (29). MAGP-1 null mice showed a lack of competition for LLC-microfibril binding, resulting in a reduced concentration of active TGF-β (28).

An additional potential regulator of TGF-β activity upstream of elastin transcription is the matricellular protein emilin-1, which associates with elastic fiber components (30) and inhibits activation of all three pro-TGF-β molecules as a regulatory mechanism for blood pressure (31, 32).

### Elastin Post-translational Modification

Elastic recoil is essential during aortic development and physiological blood flow. A murine vSMC lysyl oxidase (LOX) overexpression model resulted in more organized elastic fibers, stronger collagen assembly, and enhanced fibulin-5 production (33); however, an accompanying local H_2_O_2_ concentration increase was seen, suggesting LOX is a source of oxidative vascular wall stress. LOX knockout mice are prone to thoracic aortic aneurysm and dissection and die soon after birth, with a 60% reduction in elastin crosslinks observed in aortic tissue (34), underscoring LOX inactivation as a key prognostic indicator for aneurysm and dissection prognosis. MAGP-1 and fibronectin both repress endothelial cell LOX deposition (35), which is a secondary but significant LOX source within the vascular wall.

### Elastic Fiber Assembly

Fibrillin-1 and fibrillin-2 are both important for elastic fiber development and maintenance of a healthy vasculature. Fibrillin-2 displays preferential accumulation in elastin rich areas (36) while fibrillin-1 is more widely expressed, making it unsurprising that Marfan Syndrome patients have a wide spectrum of pathologies (37). Fibrillin-1 (38) and fibrillin-2 (39) deficient mice both die soon after birth due to aortic rupture, although qualitative differences were observed in the medial wall between the two forms of fibrillin deficiency. One theory for the different modes of action of the fibrillin isoforms is that initial fibrillin-2 involvement aids aortic matrix stability, but continued fibrillin-1 expression is essential for maturation and post-neonatal vascular function (39). This suggests that vascular engineers concerned with repair modulation must give equal attention to the initial embryonic role of fibrillins as well as adult phenotypes in order to achieve a stable engineered vessel.

Fibulin-2, a protein that binds extracellular ligands, is thought to provide redundancy with fibulin-1 (which interacts with elastin precursor tropoelastin), since fibulin-1 knockout mice produce vessels with viable elastic fibers (40). Fibulin-2 interacts with virtually all elastin precursors [though it is not associated with tropoelastin deposition by fibroblasts (41)] and is expressed in basement membrane of heart, with an enhanced role during development (42). Fibulin-2 has the highest elastin-binding affinity of the fibulins and interacts with fibulin-5 to form the elastic lamina by directing elastic fiber microassembly during development and after injury (43). Fibulin-5 is itself an elastin binding protein involved in primary organization and assembly of elastic fibers (44). In a mouse fibrosis model, loss of fibulin-5 resulted in a reduction in aortic stiffness, possibly halting the stiffness-induced local inflammatory response and deposition of ECM (45). Based on the importance of the fibulins, a calcium-dependent elastin binding protein structure domain mimicking aspects of the fibulin family could be replicated to develop a therapeutic to aid in elastin assembly (46) or interrupt the pro-fibrotic feedback loop.

Fibulin-1 and aggrecan content within the aortic wall have recently shown key roles in age-related aortic stiffening (47), with significant deposits of aggrecan and versican accumulation found during proteomic analysis of human thoracic aortic aneurysm and dissection samples (48) due to either increased synthesis or decreased proteolytic turnover. Given high stiffening conditions and predisposition toward dissection and aneurysm, fibulin-1, aggrecan, and versican present as a potential biomarker combination for aneurysmal risk evaluation.

## Matricellular Proteins That Limit Thrombosis

In large diameter vascular grafts, such as those used to repair the thoracic aorta, synthetic materials have shown great success. However, when the same synthetic materials (e.g., PTFE) have been used to make small diameter grafts (< 5 mm), thrombosis has been a major failure mechanism (49). Indeed, chemical incorporation of non-thrombogenic coatings (50) and seeding of cells that secrete pro-fibrinolytic factors (51) have been used by engineers to reduce thrombotic failure. The size of the vessel has a clear inverse relationship with the tendency to occlude, which results from the activation of thrombin and platelets (52). Upon activation of thrombin, fibrinogen in serum is converted to fibrin, which forms an insoluble fibrous mesh. Activated platelets adhere to the vascular wall and fibrin, and signal to circulating cells. The net effect of these two activation events is the formation of a thrombus loaded with fibrin and platelets that eventually occludes the small vessel. In this section we will examine how matricellular proteins regulate thrombin activation, ensuing fibrin homeostasis, as well as platelet activation in order to assess new anti-thrombotic engineering strategies.

### Thrombin Activation

Thrombosis occurs when prothrombin in the blood is cleaved and thereby gains enzymatic activity. Thrombin activation can occur through one of two distinct pathways. The extrinsic pathway is initiated by trauma and is the primary initiator of the thrombosis cascade, while the intrinsic pathway is initiated through complex formation on collagen and functions as an amplification method for homeostasis. Both pathways result in downstream activation of Factor X and subsequent thrombin activation, but the intrinsic pathway is often associated with inflammatory responses following biomedical material implantation. In either case, activated thrombin cleaves its substrate, fibrinogen, which releases fibrinogen degradation products and initiates fibril formation.

The matricellular protein vitronectin binds to plasminogen activator inhibitor 1 (PAI-1), inhibiting downregulation of PAI-1 via its receptor, LRP (53). Vitronectin is co-released with PAI-1 as a counterbalance mechanism during platelet activation and incorporates into the clot where it can modulate PAI-1 activity (54).

Fibulin-1, a fibrinogen binding blood-borne matricellular protein, displays a characteristic overlapping pattern with fibrinogen within atheromatous regions displaying fresh thrombi. This suggests that fibulin-1 correlates with thrombotic aspects of atherosclerosis and on-site thrombus formation, which serve as the leading side-effect of vascular therapies (55).

### Platelet Activation

Platelet activation is a key companion of thrombosis. Platelets interact with the vessel wall by first “rolling” along the surface, then attaching and spreading through integrin-mediated interactions. In order to activate, platelets must bind to exposed extracellular matrix, generally via the interaction of integrin on the cell surface with type I collagen.

Matricellular proteins in the thrombospondin (TSP) family (of which there are five known members) have key regulatory roles for both platelet recruitment and platelet aggregation. TSP-1 regulates platelet recruitment and anti-ADAMTS13 antibodies within stimulated endothelium (56). TSP-1 also stimulates platelet aggregation by blocking the antithrombotic activity of nitric oxide (NO)/cGMP signaling, resulting in vessel occlusion if it occurs in vascular engineered settings like balloon angioplasty, stent expansion, or therapeutic delivery (57). TSP-1 and CD36 have a co-dependent effect on platelet adhesion and collagen-dependent thrombus stabilization, with CD36 serving in an anchoring role in a TSP-1 knockout model (58). Emilin-2 regulates platelet activation/contractility and aggregation via adenosine diphosphate, collagen, and thrombin; emilin-1 also promotes platelet aggregation but in contrast reduces clot retraction (59). Both members of the emilin family therefore represent key molecules for platelet regulation.

Many alterations of vascular hemostasis have been identified in mouse models of matricellular protein deficiency. Notably, phenotypes of altered hemostasis have been difficult to identify because they are only revealed in response to injury. Mice deficient for either TSP-2 or MAGP-1 exhibit bleeding diathesis; in the case of the latter, this phenotype can be rescued with intravenous injection of recombinant protein (60). Lack of MAGP-1 alters the morphology of the thrombus, which is slow to permanently establish itself in the site of vessel damage (60, 61). The phenotype of TSP-2 deficient mice is inherent to the extracellular matrix. When fibroblasts from TSP-2 deficient mice are used as the source of a cell derived matrix, this matrix resists thrombosis compared to matrix synthesized by wild-type fibroblasts (62). Current vascular engineering strategies are exploiting this cell-derived matrix as an anti-thrombotic coating (63).

## Matricellular Proteins That Support Endothelial Formation and Maintenance

Physiological control of thrombosis is primarily performed by the endothelium lining the luminal surface of a blood vessel. To mimic the native state, an implanted vascular graft should ideally be “pre-coated” with a permanent, antithrombotic lining; unfortunately, this is rarely a feasible strategy. In contrast, a vascular graft that encourages recellularization by endothelial cells (ECs) from the host, and treatment with anticoagulants during this recellularization period, is more often used. The design parameters responsible for proper formation of a new endothelial lining are endothelial cell adhesion, migration, proliferation, and survival. In this section, we will illustrate how these functions are modulated by matricellular proteins *in vitro* and *in vivo*.

### Endothelial Cell Adhesion and Migration

CCN1 binding with α_v_β_3_ and α_II_β_3_ integrins promotes both adhesion and migration of ECs (64). A dose-dependent adhesion enhancement of ECs was also shown using CCN2 within a 2D *in vitro* model (65). CCN1 also enhances VEGF signaling by promoting VEGF production (66) and enhancing VEGF-R2 phosphorylation (67). Together, enhanced EC tubule formation (68, 69) and nitric oxide production (70) are observed with CCN1 treatment. Additionally, CCN3 has been shown to stimulate EC adhesion and migration in an α_v_β_3_ / α_5_β_1_ dependent manner (71). MAGP-2 antagonizes Notch signaling to promote cell sprouting and EC migration (72, 73). Initial formation of endothelial tubes is also promoted by SPARC, enabled primarily through TGF-β1 inhibition and preceded by pericyte migration (74).

Vitronectin, a serum glycoprotein, is found at high concentrations to promote differentiation of EC/SMC phenotypes (75). ECs bind to vitronectin via heparin sulfate proteoglycans (76), leading to EC spreading (77). α_v_β_3_ integrin binding has shown to decrease cellular motility *in vitro* on HUVECs in vitronectin coated flasks (78), and can be a target for vitronectin-focused vascular engineering. Thrombogenic PAI-1, co-released with binding target vitronectin, induces HUVEC dysfunction and procoagulant states through mast cell exosome signaling (79), underscoring vitronectin and PAI-1 as EC-linked antithrombogenic vascular engineering targets.

The proteoglycan decorin regulates EC migration and adhesion indirectly, by binding to the receptors for EGF (80, 81) and IGF (82), thereby attenuating downstream Akt and MAPK signaling. An engineered decorin mimic has also been shown to directly promote both endothelial migration and proliferation (83). Lumican, another proteoglycan, inhibits EC migration by interfering with p38 MAPK signaling (84).

TSP-1 has a generally anti-angiogenic effect on ECs (85), mediated by CD36 (86). Similarly, TSP-2 is anti-angiogenic as TSP-2 knockout mice have shown increased angiogenesis (87) and impaired von Willebrand Factor accumulation on secreted ECM (62). In double TSP-1/TSP-2 knockout animals, neoangiogenesis is mediated by MMP9 activation (88). In contrast to the anti-angiogenic activities of TSP-1 and TSP-2, TSP-4 is required for proper increased adhesion, migration, and proliferation of ECs (89, 90). CD47, an essential TSP-1 receptor (91), can mediate TSP-1 function and is therefore another useful target for controlling angiogenesis. CD47 function is altered by a lipid environment (92), so angiogenesis can be manipulated by controlling the VLDL receptor in endothelial cells and thus decrease Akt and MAPK phosphorylation (93). From an engineering perspective, TSP-2 knockout mouse dermal fibroblast secreted ECM was used to coat decellularized rat aorta, resulting in an anti-thrombotic and pro-migratory vascular graft that improved EC recruitment and decreased failure rate following interpositional rat abdominal aortic implantation (63).

### Endothelial Cell Proliferation and Survival

CCN1 enhances proliferation of endothelial cells by augmenting bFGF signaling (94), and also promotes endothelial cell survival (64). CCN2's effect is somewhat more complex, as it contains modules that can either enhance (95) or decrease (96) proliferative signaling. CCN3 overexpression suppressed VCAM-1 (which supports adhesion) and normal CCN3 expression reduced monocyte adhesion. TNX increases EC proliferation through interactions with VEGF (97). Fibrillin-1 RGD-containing sequences have the ability to stimulate EC proliferation *in vitro* (98). TSP-2 alone inhibits growth-factor induced microvascular EC proliferation in a caspase-independent manner (99). SPARC reduces VEGFR-1 after injury, resulting in anti-angiogenic activities early in the development cascade and opening the door for VEGF-A targeted therapeutics that route through VEGFR-1 pathway (100).

## Matricellular Proteins That Recruit and Regulate Vascular Smooth Muscle Cells

As described above, a native vessel must possess the mechanical strength and resilience to survive the dynamic forces it will encounter over the course of a human life. In turn, an engineered vessel must be able to outlast the patient; in the case of pediatric patients, grafts must also grow with them. Vascular smooth muscle cells (vSMCs) are essential in the maintenance of vascular walls *in vivo*, and thus are prime targets for vascular engineers.

For the purposes of this review, we will highlight two main functions of vSMC in small vessels. First, vSMC are responsible for the synthesis, alignment, and maintenance of elastin during development to provide effective passive recoil in response to hemodynamics and local tissue deformation. vSMC also can potentially synthesize new elastin in response to damage; however, it appears that adult vSMC are limited in their elastin expression at the transcriptional level. Second, vSMC cooperate with EC to regulate vascular tone. EC receive signals from the circulation that stimulate production of the second messenger nitric oxide (NO), inducing vSMC relaxation and vessel dilation. In contrast, agents such as angiotensin II act directly on vSMC, causing the cell to contract and the vessel to constrict.

It is clear, then, that the presence of functional vSMC in a mature vascular graft would be ideal. As was the case for ECs, implanting a graft which already contains vSMC is rarely feasible, and more often recellularization by host cells is used. Therefore, in this section we will examine how matricellular proteins modulate vSMC adhesion, migration, proliferation, and survival, as well as vSMC function in the context of neointima formation.

### Smooth Muscle Cell Adhesion and Migration

Angiotensin II promotes atherosclerosis by inducing the adhesion and migration of vSMCs, and is mediated by osteopontin using putative binding site miR181a in its inhibition (101). MAGP-1 is dependent on MAGP-2 in binding/activating Notch 1, which activates Notch signaling and modulates homeostasis during aortic SMC spreading (72).

Fibulin-5, in addition to its well-documented role on elastogenesis, is the primary mediator of uPA-driven migration of vSMCs (102). The mechanism for this appears to be that fibulin-5 binding facilitates activation of uPA, which then activates plasmin to cleave the portion of fibulin-5 that binds β_1_ integrin, driving cell migration. This action increases vSMC remodeling and vascularization after injury and antagonizes angiogenesis by inducing TSP-1 and antagonizing fibronectin receptors (102, 103). Galectin-1, a glycan-binding protein primarily involved within the anti-inflammatory cardiac tissue response to acute myocardial infarction, has been shown to restrict vSMC motility and modulates focal adhesion turnover on fibronectin, via *in vitro* assays using galectin-1 knockout mouse SMCs (104).

### Smooth Muscle Cell Survival, Proliferation, and Neointimal Formation

In post-injury vasculature, galectin-1 is upregulated within proliferating SMCs, and in confirmatory studies an engineered galectin-1/GST fusion protein is able to drive SMC proliferation (105). Taken with its effects on increasing SMC proliferation and restricting SMC motility (105), galectin-1 presents as a potential supplement for cardiac therapeutics post-myocardial infarction and a serum biomarker for cardiac stress (106) with a well-characterized mouse model for *in vivo* evaluations.

The CCN family of matricellular proteins have significant, yet indirect, effects aiding vascularization and angiogenesis that might be considered in engineering microenvironment applications. Full length CCN2 utilizes integrin α_6_β_1_ to promote adhesion and spreading of vSMCs (65). CCN5 also inhibits vSMC proliferation and motility without affecting apoptosis and adhesion (107, 108), and has potential to be used as an delivered therapeutic CCN2 antagonist to control vSMC migration, adhesion, and phenotype (109). CCN5's effects are modulated in a dose-dependent fashion by PDGF and TGF-β, with some possible quiescence-related SMC properties (107). A reduction in CCN2 expression was observed in Notch1 haploinsufficiency murine abdominal aortic aneurysm models, leading to a maintenance of contractile vSMC phenotype and a reduction in aortic dilation (110). CCN2 presents with a key target for SMC phenotype maintenance in vasculopathy.

CCN4 (also known as Wisp-1), is upregulated in migrating vSMC in a Wnt2-dependent manner; loss of CCN4 leads to inhibited vSMC integrin mediated migration (111). Wnt5a, which induces β-catenin signaling in mouse via vSMCs, saw CCN4 rescue VSMCs from H_2_O_2_ induced apoptosis within atherosclerotic plaque (112, 113). CCN4 was expressed within advanced human coronary artery lesions (111) but absent in Wnt5a positive intimal SMCs, indicating that CCN4 deficiency may provoke vSMC apoptosis in coronary plaques, ultimately resulting in instability. Loss of CCN4 leads to reduced intimal thickening, related to CCN4's aforementioned positive effect on vSMC migration (111).

Tenascin-C (TNC) within the context of atherosclerotic plaques promotes SMC proliferation and migration via PDGF signaling (114). Areas of human abdominal aortic aneurysm with high TNC expression by medial SMCs correlate strongly with inflammation and tissue ECM destruction (115). TNC overexpression can also result in pulmonary hypertension, and it is expressed in adventitia and media of saphenous vein grafts under above average arterial stress pressure (116). Taken together, TNC presents with a SMC-linked biomarker target to assess atherosclerosis or aneurysm pathological state.

Neointimal hyperplasia is a common complication of vascular disease intervention (117). The main target for anti-neointimal formation is SMC phenotype, given the predisposition of unregulated hyperplastic vSMC proliferation to accelerate neointimal formation. Many matricellular proteins have shown efficacy in controlling vSMC phenotype and adhesion, and therapeutics often target at upregulation of these factors. TSP-1 targets NO-mediated vSMC relaxation to increase tissue survival and facilitate tissue perfusion to preserve tissues under ischemic stress (118). vSMC are also induced to proliferate by TSP-1 (119). Fibromodulin adenovirus-mediated gene transfer inhibits restenosis in an organ culture saphenous vein graft disease model of neointimal hyperplasia, at a much greater level than decorin or beta-galactosidase gene transfer (120). Fibromodulin could be a key SLRP for study within ECM-based vein grafts.

Another ECM-related inducer of neointimal formation is fibulin-1, where expression is closely overlapped with fibrinogen. Fibulin-1 and fibrinogen bind tightly within atherosclerotic legions, but this does not hold true in ECM associated with SMC surrounding the identified lesion. Fibulin-1 is associated with atherogenesis in diabetic patients, and at high levels fibulin-1 becomes an accurate indicator of cardiovascular risk in patients prior to acute cardiovascular events, obesity, or diabetes risk markers (121). vSMCs display increased SMC contractile differentiation markers with fibulin-5 overexpression (44), and vSMC phenotype has also been regulated through fibulin-4 expression in murine models (122). Overexpression of fibulin-5 has been shown post-injury to increase SMC motility in a step to aid in vascular remodeling (102).

CCN3 exhibited a protective role within the progression of murine abdominal aortic aneurysm model (123), and inhibited neointimal hyperplasia by inhibiting vSMC migration and proliferation (124). With this background, novel CCN3-based peptides have recently been developed, aiding in the modulation of pro-fibrotic factors CCN2, PAI-1, and post-translational fibrotic collagen modification (125).

Osteopontin (OPN, also known as bone sialoprotein I, abbr. BSP-1), a matricellular protein first identified within osteoblasts, is expressed by vSMC derived foam cells after immune response, and plays a large role in the calcification of atherosclerotic lesions (126). Increased OPN expression during neointimal hyperplasia formation was seen as a major microenvironment component of human atherosclerotic plaque progression. OPN downregulates vSMC differentiation markers αSMA and calponin, transitioning from quiescent status into an unregulated proliferative phenotype (127). Galectin-1 binds to lipoprotein(a), which binds tightly to LDL and are both co-implicated in atherogenesis. Lipoprotein(a) binds poorly to tissue with galectin-1 inhibition, suggesting that lipoprotein(a) accumulation changes have an effect on atherogenesis, possibly mediated through t-antigens on lipoprotein(a) and LDL (128). A novel engineering approach modulating OPN protein expression or galectin-1 binding within atherogenic arterial walls could present as a key mediating factor for downstream neointimal hyperplasia.

## Matricellular Proteins in Vascular Tissue Engineering

To date, only a few of the matricellular proteins reviewed here have been pursued in the context of vascular tissue engineering, hence the need for future tissue engineering strategies harnessing these proteins. What follows is a brief summary of the findings from this small library of published work—note that by far the most predominant area of study has been how to encourage endothelial adhesion to biomaterials in the vascular context.

### Production of New Vascular Matrix

Mature elastic fibers that are mechanically competent are a highly sought-after component of a TEVG. Several groups have investigated methods to improve elastin content, or production, within grafts and unsurprisingly a few of these studies have harnessed matricellular proteins to do so. Versican, as explored by the Wight group, has a complex relationship with elastogenesis. Full length glycosylated versican, known as V0, negatively impacts new elastin fiber formation. However, a splice variant of versican that cannot be glycosylated, known as V3, drives new elastin formation and cross linking by SMCs (129). With this background, the Wight group collaborated with a vascular tissue engineering company, Cytograft, to explore the potential for a TEVG with enhanced elastin content. Cell sheets of SMCs retrovirally expressing V3, or control cells, were cultured for 12 weeks to form a rich cell-derived matrix, rolled on a mandrel, and allowed to fuse for 18 more weeks. During the initial cell culture period (4 weeks), not only was expression of elastin elevated at the gene level, as expected, but also fibulin-5 (130). Mature crosslinking was also elevated by V3, as well as higher mechanical strength as judged by burst strength. It should be noted that in the cell-sheet TEVG strategy, the cells are generally removed before further TEVG processing, so the initial transduction of the cells should not serve as a hurdle to final FDA approval.

An alternate method for exploring improved elastin production was published by the Suzuki group. As reported by others (131), collagen is generally a poor 3D matrix for cellular elastic fiber production. The Suzuki group had previously discovered a beneficial effect of LTBP-4 on elastic fiber assembly (132) and reasoned that it could also then encourage elastic fiber formation in collagen gels. Here, human skin fibroblasts were seeded on top of porous collagen sponges, allowing the cells to infiltrate the sponge during culture. Recombinant LTBP-4 was added to experimental cultures at a concentration of 5 μg/ml, and replaced along with media changes every week for 3 weeks. Increased LTBP-4 immunostaining was observed in the LTBP-4 treated cultures (both recombinant and native human protein), as well as improved fibrillin-1 and elastin (133). Interestingly, the benefit on elastin was also observed with a single dose of LTBP-4 at 1 week, as opposed to the 3 week treatment described above—although immunostaining for fibrillin-1 was not explored. This work was limited by lacking more rigorous analysis of the elastic fibers (such as desmosine assay) but the simple addition of recombinant LTBP-4 to culture media is more amenable to the typical tissue engineer than retroviral work, and therefore of note.

### Limiting Thrombosis

Because thrombosis can be initiated upon contact of blood with ECM such as the vascular basement membrane and internal elastic lamella, most groups have focused on endothelialization (see next section). However, in work by the Kyriakides lab analyzing a mouse model for TSP-2 deficiency, it became clear that TSP-2 deficient arteries are resistant to thrombosis for reasons linked to the ECM of the vessel wall (not just inherent to thrombus formation) (62). In a similar situation to the Wight group collaboration with Cytograft explored in the previous section, Kyriakides teamed up with Laura Niklason from the vascular tissue engineering company Humacyte to explore if a TSP-2 deficient matrix could serve as a thrombosis-resistant lumenal coating for the Humacyte graft. Modification with the TSP-2 deficient matrix had no effect on the mechanical properties of the graft, but both inhibited platelet activation *in vitro* and improved endothelialization of interpositional abdominal aortic implants after 4 weeks *in vivo* (63). They do not report an effect on patency as seen by our group when evaluating TEVGs in a similar model (51), but it could be expected that as they move to a larger animal model a patency effect could be revealed.

### Endothelial Formation and Maintenance

As discussed above, CCN1 has a pro-adhesive and pro-migratory effect on ECs. Hilfiker, Haverich, Wilhelmi, and Boer have performed collaborative work for nearly a decade using recombinant CCN1 to promote re-endothelialization of decellularized small intestine with preserved artery and vein pedicles (134), heart valves (135), and decellularized arteries (136, 137). Introduction of recombinant CCN1 (100 ng/ml overnight for the intestine and arteries, 400 μg/ml 6 h for the heart valves) was performed either by perfusion of cannulated tissue (intestines) or end-over-end incubation of the tissue and CCN1 solution (heart valves and arteries). In either case, introduction of matricellular protein to engineered tissues appears to be both simple and effective using these methods, and could be extended to other molecules.

The Chaikof group has investigated the use of elastin-like peptides (ELPs) as the basis of a new biocompatible and tunable hydrogel scaffold for vascular tissue engineering. Since the ELPs self-assemble, coacervating at a tunable transition temperature, it is relatively simple to incorporate other cargo into the gelation mixture. Two matricellular proteins have been investigated in this context—fibronectin (138) and CCN1 (139). Two methods were used for incorporation of fibronectin into ELPs. In one method, ELPs were coacervated into hydrogels, which were then incubated in a 1 mg/ml solution of fibronectin for 4 h of adsorption before genipin crosslinking of the ELPs. In the second method, ELPs and fibronectin were blended at 4°C before coacervation into hydrogels at 37°C and subsequent genipin cross-linking. While improved pro-adhesive activity toward ECs was observed with the adsorption method, both fibronectin-loaded hydrogel strategies outperformed ELPs alone. Other beneficial outcomes included improved spreading and migration of ECs (138). A distinct strategy was used to incorporate beneficial CCN1 activities into ELP hydrogels—instead of adding recombinant protein to the gelation mixture the peptides themselves were engineered to contain a biologically active sequence derived from CCN1. Specifically, this 20-residue sequence, known as the V2 domain, is critical for integrin α_v_β_3_-dependent binding of ECs to CCN1 (140) and therefore represents a new cell-interaction site for engineered biomaterials. Ten repeats of the V2 sequence were introduced into the standard triblock structure of the ELP; hydrogels containing V2 had improved EC adhesion that could be blocked by soluble V2 peptide or an antibody that interferes with α_v_β_3_ function (139). As was the case with fibronectin-functionalized ELPs, beneficial outcomes included improved migration and spreading of ECs.

In a similar vein to the Chaikof CCN1 experiments, the Heath lab has recently published work exploring the synergistic EC-adhesive effect of traditional integrin-binding RGD peptide along with peptides used for binding to syndecans −1, −2, and −4 (141). In this case, the hydrogel system used was polyethylene glycol (PEG), a common biomaterial explored in tissue engineering; within the PEG network, RGD peptide and/or one of two different syndecan-binding peptides were conjugated. While RGD and syndecan-binding peptides did promote EC adhesion beyond their respective controls (RGE and a scrambled syndecan-binding peptide) the combination of RGD and syndecan peptide at ratios of 1:1 or 3:1 promoted superior adhesion. Interestingly, this worked whether the two peptides were in separate PEG chains (nanoclusters) or in the same PEG chain (heteroclusters). Beyond adhesion, proliferation was also accelerated with the peptide mixture, vs. single peptides. Perhaps the most astonishing finding of this work was revealed with flow. ECs adherent to single peptides stayed adherent under 10 h of flow (3.1 dyn/cm^2^) but did not form proper focal adhesions or align to flow—in contrast ECs adherent to the peptide mixture formed prominent focal adhesions, stress fibers, and re-aligned with flow (141). A recent review on syndecans has explored their role in the context of regeneration and their potential to regulate stem cell function, though not specifically in the context of vascular tissue engineering (142).

As discussed earlier, small diameter PTFE grafts typically undergo thrombotic failure. While seeding of ECs has been explored, they are generally unable to remain within the graft long term and ultimately the graft continues to fail. The Flugelman group has explored the use of fibulin-5, in addition to VEGF, as a method to retain ECs (143). Specifically, ECs were retrovirally transduced to express both fibulin-5 and VEGF, and then dually transduced, GFP transduced, or naïve ECs were seeded into PTFE scaffolds for *in vivo* testing. Both short term (2 weeks, interpositional) and middle term (3 or 6 months, end-to-side) carotid artery implantations were performed in sheep. A substantial benefit was observed using the fibulin-5/VEGF ECs with respect to patency, vs. GFP transduced and naïve EC controls. While there are some limitations to the study concerning a lack of evaluation of fibulin-5/elastin deposition in explants or a fibulin-5 only control, it is an exciting foray in the context of matricellular proteins in vascular tissue engineering.

An alternate method for inducing endothelialization of vascular scaffolds is to recruit circulating endothelial progenitor cells (EPCs). Work by the Schenke-Layland has explored the use of decorin as a ligand to attract EPCs from the circulation and thereby quickly coat an implanted scaffold with a “pre-endothelium.” An electrospun PEGdma-PLA scaffold, meant to serve as a heart valve equivalent, was coated with recombinantly expressed decorin, stromal-derived factor 1, or were left uncoated. Using a dynamic *in vitro* cell capture system, human and mouse EPCs were exposed to the scaffolds for 24 or 48 h, respectively. Decorin and stromal-derived factor 1 both led to higher capture of EPCs compared to bare scaffolds, in addition the captured cells proceeded to form focal adhesions at the conclusion of the incubation period (144). While it should be noted that this has not yet been tested with whole blood, or under flow, the system described here could be a novel method for endothelializing an a cellular implanted scaffold.

### Smooth Muscle Cell Recruitment

Aside from the above studies regarding new elastin production by SMCs, little has been published regarding the role of matricellular proteins in SMC recruitment for vascular tissue engineering. One study of note, however, investigated the chemical conjugation of whole fibronectin, not just the RGD peptide, to porous poly(carbonate) urethane scaffolds (145). Considering that fibronectin is a 500 kD protein, this conjugation was not trivial. In this work, scaffolds functionalized with full-length fibronectin encouraged SMC recruitment to a depth of up to 300 μm within the material; non-functionalized scaffolds only supported SMC growth on the surface. Therefore, fibronectin incorporation into porous scaffolds for vascular tissue engineering may promote host cell SMC recruitment.

## Summary

Much of the groundwork for matricellular protein-based protein engineering has been laid by the two decades of biological studies reviewed here. The goal of this review is to get the results of these studies into the hands of bioengineers developing new cardiovascular technologies, and to spark interdisciplinary discussions that can lead to novel solutions for cardiovascular disease. A summary of the biological effects of each individual matricellular protein is shown in Table 2. Admittedly, the knowledge base reviewed here is constantly evolving, but the intent is that established collaborations will then stay informed about their protein(s) of interest. Another caveat is that regeneration of the vasculature likely involves the cooperation of several of these factors in concert, which may not have a simple summative effect compared to the individual activities.

**Table 2:** Summary of biological effects for each matricellular protein discussed in this review (see respective sections for details and references).

**Protein name**	**Summary of effects**
Aggrecan	Potential biomarker for aneurysm risk
CCN1	Stabilizes collagen fibers, promotes adhesion and migration of ECs
CCN2	Promotes collagen transcription, EC adhesion, and SMC adhesion/spreading
CCN3	Reduces EC adhesion and inhibits SMC proliferation
CCN4	Promotes SMC migration, inhibits SMC apoptosis in atherosclerotic plaque
CCN5	Antagonizes CCN2, inhibits SMC proliferation and motility
Decorin	Increases density of collagen bundles, associated with aneurysm risk
Emilin-1	Inhibits TGF-β activation, binds to elastic fiber microfibrils, promotes platelet aggregation and inhibits clot retraction
Emilin-2	Binds to elastic fiber microfibrils, promotes platelet aggregation and clot retraction
Fibrillin-1	Regulates TGF-β activation, scaffold for elastic fibers, promote EC proliferation
Fibrillin-2	Regulates TGF-β activation, scaffold for elastic fibers
Fibromodulin	Inhibits SMC proliferation/restenosis
Fibulin-1	Associated with thrombosis, aneurysm, and cardiovascular disease risk
Fibulin-2	Directs elastic fiber assembly
Fibulin-5	Directs elastic fiber assembly, promotes SMC migration and contractility
Galectin-1	Promotes SMC spreading and adhesion, reduces motility, mediates atherogenesis
LTBP-1 and−4	Inhibit TGF-β activation, binds to elastic fiber microfibrils
Lumican	Promotes LOX activity, inhibits EC migration
MAGP-1	Modulates sequestration of TGF-β, integral to elastic fiber microfibrils, required for proper thrombosis
MAGP-2	Integral to elastic fiber microfibrils, inhibits EC Notch signal to promote migration
OPN	Promotes proliferation of SMCs and calcification of atherosclerotic lesions
SPARC	Promotes collagen bundling, EC migration
Syndecan-4	Promotes collagen post-translational processing
TNC	Promotes SMC proliferation and migration
TNX	Promotes collagen post-translational modidication, EC proliferation
TSP-1	Promotes platelet aggregation, inhibits EC angiogenesis, promotes SMC proliferation
TSP-2	Required for proper thrombosis, inhibits EC proliferation and angiogenesis
Versican	Associated with aneurysm risk, inhibits elastic fiber formation
Vitronectin	Via PAI-1 reduces thrombus removal, promotes EC adhesion, and spreading

Matricellular proteins can be used to enhance production of new collagen and elastin, the primary contributors to vascular strength and resilience. Much of the literature on collagen transcriptional regulation can be informative for future engineering of collagen-rich tissues, and reciprocally informative for strategies to reduce graft fibrosis or the foreign body response to cardiovascular devices. Understanding how matricellular proteins guide collagen bundling can be used to inform future collagen-based biomaterials. Notably, new elastin production by adult cells has been something of a holy grail in the field of vascular regenerative medicine, with several recent studies in the field of aneurysm therapeutics focused on production of this important molecule (146, 147). Including matricellular proteins such as members of the fibulin family in future cardiovascular tissue engineering strategies will be critical for achieving fully mature, mechanically sound, elastic fibers. Understanding how these elastin chaperones work will also be important for designing devices to help patients with deficiency in these important proteins.

A key limitation of synthetic small diameter grafts is the occurrence of thrombotic occlusion; as explored here it has become clear that matricellular proteins such as members of the TSP family can have a significant effect on how thrombosis occurs. The work by Kyriakides et al. demonstrating thrombosis resistance in the absence of TSP-2 is particularly striking in this regard (62, 63). Regulation of thrombosis by matricellular proteins can be due to inhibition of thrombin activation, platelet activation, or both. While the focus in this review has been on thrombotic failure in vascular grafts, these findings also have implications for the design of cardiovascular devices with lower risk of embolism and stroke.

In a tissue-engineered graft, formation of an intact, thromboresistant endothelium is critical to enable prolonged function. In development and maintenance of the endothelium, matricellular proteins found in both the circulation and the underlying basement membrane have critical roles. Not only are these proteins important to support endothelial adhesion, they also are important for promoting survival of adherent ECs, which will be critical for a small-diameter engineered graft. Maintenance of ECs in a non-pathologic phenotype is also an area of interest in vascular engineering, so understanding the role of matricellular proteins on EC differentiation state will also be critical for future device design.

Finally, an understanding of the effects of matricellular proteins on vascular SMCs will be beneficial for future vascular engineering, since SMCs not only are critical for production (and turnover) of new vascular matrix in the graft context but also perform multiple roles in normal vascular physiology. Implanted cell-free grafts must support survival and ingrowth of host SMCs to better mimic the native artery. Matricellular proteins have the ability to promote both of these outcomes. In addition, a common failure method for the most used graft in coronary bypass operations—an autologous saphenous vein graft—is neointimal formation and occlusion due to excessive proliferation of SMCs. Control of SMC overproliferation by peptides derived from matricellular proteins could replace conventional drug-eluting stent technologies, and also be used to augment current vascular graft strategies.

In summary, we would propose that the use of matricellular proteins and matricellular-derived peptides could serve as a paradigm shift in the field of vascular tissue engineering.

## Author Contributions

Conception of this article was primarily by JW. Literature research was performed by AR and JW. Writing and critical revision were performed by AR, DV, and JW.

### Conflict of Interest Statement

The authors declare that the research was conducted in the absence of any commercial or financial relationships that could be construed as a potential conflict of interest.
